# A gene expression study of ornamental fin shape in *Neolamprologus brichardi*, an African cichlid species

**DOI:** 10.1038/s41598-017-17778-0

**Published:** 2017-12-12

**Authors:** Ehsan Pashay Ahi, Florian Richter, Kristina M. Sefc

**Affiliations:** Institute of Zoology, University of Graz, Universitätsplatz 2, A-8010 Graz, Austria

## Abstract

The diversity of fin morphology within and across fish taxa offers great, but still largely unexplored, opportunities to investigate the proximate mechanisms underlying fin shape variation. Relying on available genetic knowledge brought forth mainly by the comprehensive study of the zebrafish caudal fin, we explored candidate molecular mechanisms for the maintenance and formation of the conspicuously elongated filaments adorning the unpaired fins of the East African “princess cichlid” *Neolamprologus brichardi*. Via qPCR assays, we detected expression differences of candidate genes between elongated and short regions of intact and regenerating fins. The identified genes include skeletogenic and growth factors (*igf2b*, *fgf3*, *bmp2* and *bmp4*), components of the WNT pathway (*lef1*, *wnt5b* and *wnt10*) and a regulatory network determining fin ray segment size and junction (*cx43*, *esco2* and *sema3d*), as well as other genes with different roles (*mmp9*, *msxb* and *pea3*). Interestingly, some of these genes showed fin specific expression differences which are often neglected in studies of model fish that focus on the caudal fin. Moreover, while the observed expression patterns were generally consistent with zebrafish results, we also detected deviating expression correlations and gene functions.

## Introduction

The phenotypic diversity of fish is outstanding among vertebrates. One part of this immense diversity is variation in fin shape, which often distinguishes even closely related species (e.g. *Xiphophorus* platyfish and swordtails, *Bodianus* lipfish, various gourami and cichlid genera). Although fin shape is connected with swimming performance^1^, fins also serve important signal functions in competitive and sexual interactions. Both courtship and aggressive displays typically involve the presentation of erected fins, and the importance of fin shape in mate choice and male-male competition^2–4^ suggests that various selection pressures play a role in fin shape diversification.

The developmental mechanisms regulating fin growth and shape have mainly been studied in the zebrafish caudal fin^5^. The capability of fish to fully regenerate amputated fins makes them an ideal model in regeneration biology, not least because of the high degree of conservation in developmental patterning between fish fins and the appendages of other vertebrates^5–7^. The teleost fin is supported by segmented fin rays, which consist of an inner core of mesenchymal cells, vessels and nerves, shielded by bone (hemirays) and covered with epithelium. The fin rays grow by the addition of bony segments at their distal end, and altered regulation of fin ray growth, including cellular proliferation, differentiation and survival, can be an important source of fin shape variation^8^. Regeneration studies showed that after amputation, regeneration of the fin tissue is initiated by the establishment of highly proliferative tissue (blastema) immediately distal to each truncated fin ray. The blastema continues to elongate at the distal edge while the proximal part of the new tissue re-differentiates into the mature fin structures until the original size is restored^5,9^. At the molecular level, components of various developmental pathways, such as WNT, FGF, Hedgehog and retinoic acid (RA) signalling, as well as epigenetic, skeletogenic and structural remodelling factors have been identified to orchestrate both ontogenetic and regenerative fin growth^8–10^. Recently, candidate effectors (gene products and metabolites) of positional memory, a prerequisite for the regeneration of the proper appendage size and pattern, were described in the zebrafish caudal fin^11^.

**Figure 1:**
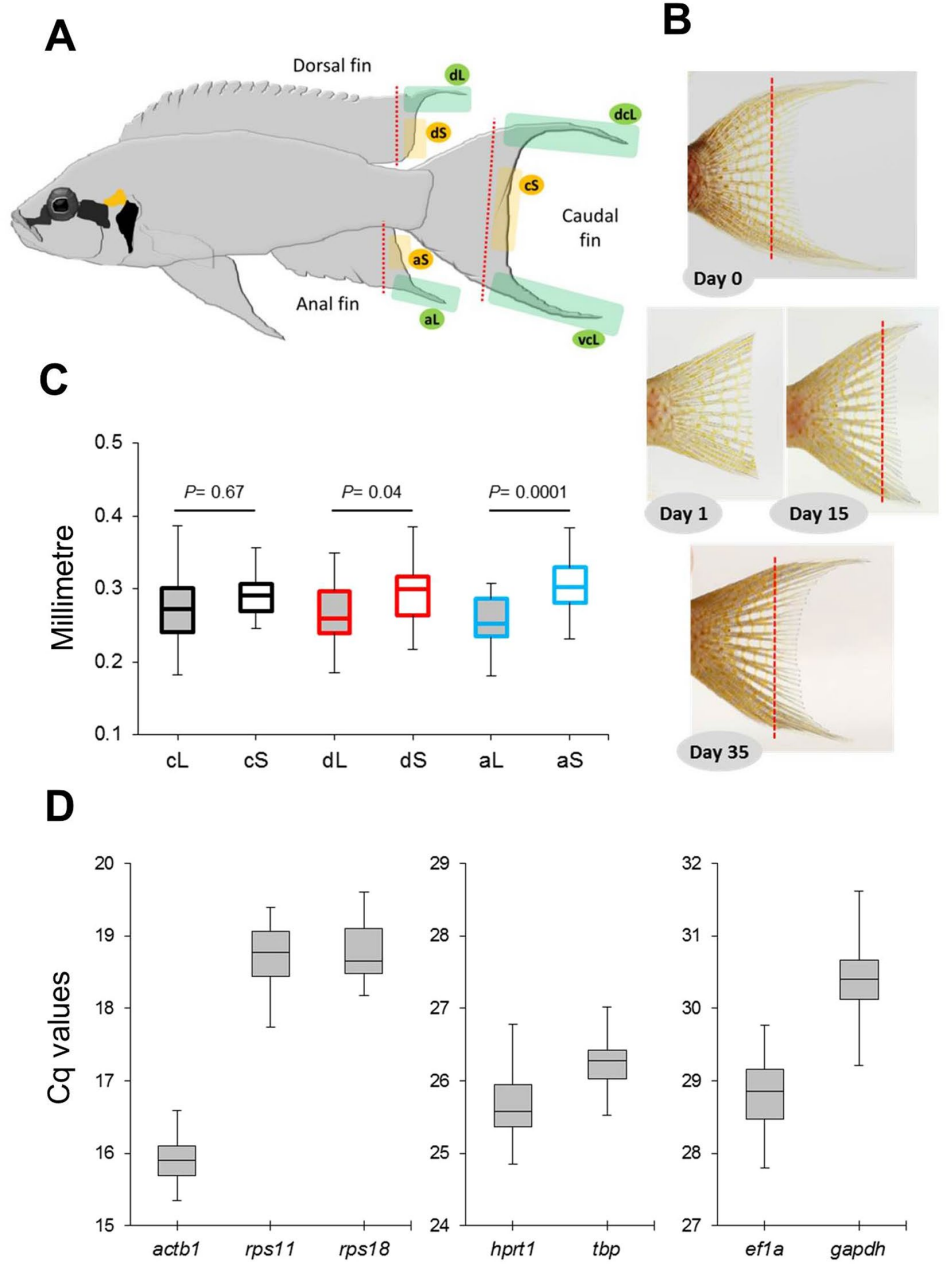
Fin dissection in *Neolamprologus brichardi*, fin ray segment length measurements and expression analysis of candidate reference genes. (**A**) An adult Lake Tanganyika cichlid fish, *N*. *brichardi*, displays regional elongation of its unpaired fins. Fins were amputated along the dashed red line, and tissue samples were taken from the elongated fin parts (green shadows) and from the short regions (yellow shadows) of the fins. (**B**) Biopsies were taken from the original (day 0) and regenerated (days 15 and 35) fin tissue, as shown here for the caudal fin (photos by Wolfgang Gessl (www.pisces.at)). The slight curve of the distal edge of the caudal fin on day 1 is due to incomplete spreading of the fin. (**C**) Fin ray segment length in L and S regions of each fin. P-values were obtained by linear mixed models. (**D**) Expression profiles of candidate reference genes in raw Cq values for all samples (all anal, dorsal and caudal fin samples of 3 cutting steps from 3 biological replicates). The middle line denotes the median and boxes indicate the 25/75 percentiles.

Contrasting with the extensive work in fish models (mainly zebrafish), little use has so far been made of the natural diversity among fishes^12–14^. The resources created in zebrafish open countless possibilities to investigate genes, regulatory networks, signalling cascades, etc., for their role in fin shape diversification and to test for molecular convergence of fin shape determination across species. Moreover, as most research has focused on the caudal fin because of its particularly fast regeneration^5^, it is not yet clear whether the mechanisms controlling fin growth are consistent across fin types. In the current study, we take advantage of the fin shape characteristics of an East African cichlid fish *Neolamprologus brichardi*, namely the long filaments adorning the unpaired fins of the adult fish (Fig. 1A). We hypothesized that the formation of this local outgrowth is influenced by genes involved in fin ray organization and growth, and predicted differences in gene expression levels between elongated and short fin regions during the maintenance of the phenotype in adult fins and/or during their regeneration. Importantly, our study species allows us to compare gene expression levels between adjacent normal (short) and elongated regions within a fin type, and then compare patterns of gene expression differences across fin types. Furthermore, the availability of an annotated transcriptome^15^ for *N*. *brichardi* facilitates the utilisation of zebrafish genetic resources. The 40 candidate genes investigated in this study belong to different signaling pathways and functional groups (e.g. skeletogenesis, extracellular matrix formation and WNT signalling) associated with ontogenetic and regenerative fin growth in teleost fish, mainly zebrafish (Table 1). Comparisons between elongated and short fin regions and between regeneration stages of each fin type identified a number of differentially expressed genes, some of which showed significant expression correlations. With this work, we provide the first investigation of the molecular mechanisms underlying ornamental outgrowth of unpaired fins in a cichlid fish and lay the foundation for further comparative studies within and beyond the highly diverse cichlid family.

**Table 1:** Selected candidate target genes involved in fin development, morphogenesis and regeneration.

Gene Symbols	Related function	Fin development/morphogenesis	Fin regeneration	References
*bmp2/4/8a*, *sp7*	BMP pathway ligands and a bone specific TF involved in induction of osteoblast differentiation, blastemal cell proliferation and skeletogenesis	+	+	^8,9,23,32–35,66^
*fgf3/10a/20a*, *fgfr1a*, *pea3*	FGF pathway components involved in controlling blastemal cell proliferation and survival, fin epithelium formation and homeostatic regeneration	+	+	^8,9,30,38,39,48,67–70^
*cyp26a1*, *raldh2*, *crabp2*, *rarg*	RA pathway components involved in controlling/ inhibiting blastema formation and basal epidermal layer during fin development and regeneration	+	+	^8,9,24,35,71–73^
*wnt5b/10a*, *lef1*, *axin1*	WNT pathway components orchestrating different aspects of blastema and basal epidermal formation in fin	+	+	^8–10,32,36–39^
*shh*, *ptch1/2*	Hh pathway components involved in patterning and growth of fin ray skeleton during development and regeneration	+	+	^8,33,35,38,74^
*hdac1*, *hspa9*, *smarca4*	Components of epigenetic gene regulation implicated in fin regeneration and controlling blastema cell proliferation	?	+	^10,75^
*evx1*, *cx43*, *esco2*, *hapln1a*, *sema3d*	Components of an interconnected regulatory network involved in formation, growth and regeneration of fin ray segments and joints	+	+	^40–44,46,76^
*mmp9*	A matrix metalloproteinase participating in the reconfiguration of tissues during fin regeneration	+	+	^10,50^
*dlx5a*	A homeodomain transcription factor required for both paired fin outgrowth and unpaired fin morphogenesis	+	+	^10,77^
*igf2b*	An insulin growth factor required for the induction of the blastemal and wound epidermal markers	?	+	^29^
*junb*	A component of Ap-1 transcriptional complex; its prolonged induction is required for fin regeneration	?	+	^10,78^
*klf2*, *rack1*	Two novel genes with a role in regional extension of fins in swordtail fish	+	?	^12^
*mps1*	A kinase required for the mitotic checkpoint and proliferation of proximal blastemal cells during fin regeneration	?	+	^79^
*msxb*	A transcriptional repressor specifying the boundary of blastemal cell proliferation and directing fin regenerative outgrowth	+	+	^48,80^
*notch1a*	A Notch pathway receptor maintaining blastema cells in a plastic, undifferentiated and proliferative state	?	+	^81^
*rpz*	A gene involved in symmetrical and allometric fin outgrowth by affecting the number of fin ray segments	+	?	^82,83^
*sdf1a*	A stromal cell-derived alpha chemokine essential for epidermal cell proliferation during blastema formation	?	+	^84,85^
*yap*	An effector of the Hippo pathway regulating regenerative growth by balancing cell density and cytoskeleton activity	?	+	^86^

## Results

### Morphological characterization

The outgrowth of fin filaments, *i*.*e*. the elongations of the unpaired fins, was not associated with increases in the length of fin ray segments in these regions. In contrast, segments in the elongated fin regions were slightly shorter than in the short regions (Fig. 1C). We conclude that the elongation of fin rays is due to increases in the number, but not length, of fin ray segments.

### Validation of reference genes

Gene expression analyses by qPCR rely on the validation of stably expressed reference genes^16^, and depending on species, tissue and experimental conditions, the expression stability of reference genes can be variable^17^. In order to identify suitable reference genes for our study, we examined the expression of 7 reference gene candidates in cDNA of the tissue samples from the intact and regenerating fins. The expression levels of these candidate genes from highest to lowest (lowest to highest Cq) are *actb1* > *rps11* > *rps18* > *hprt1* > *tbp* > *elf1a* > *gapdh* (Fig. 1D). Based on standard deviations of Cq values and the three algorithms implemented in BestKeeper, geNorm and NormFinder, *rps18* and *actb1* consistently achieved top ranks for expression stability (Table 2). Consequently, expression of *rps18* and *actb1* was used to normalize target gene expression for quantitative comparisons between fin regions.

**Table 2:** Ranking and statistical analyses of candidate reference genes using BestKeeper, geNorm and NormFinder.

BestKeeper				geNorm		NormFinder	
Ranking	SD	Ranking	r	Ranking	M	Ranking	SV
*actb1*	0.265	*rps18*	0.95	*rps18*	0.368	*rps18*	0.147
*tbp*	0.289	*actb1*	0.855	*actb1*	0.391	*actb1*	0.161
*rps18*	0.336	*hprt1*	0.762	*tbp*	0.425	*tbp*	0.224
*rps11*	0.339	*rps11*	0.759	*hprt1*	0.437	*rps11*	0.234
*hprt1*	0.349	*tbp*	0.714	*rps11*	0.452	*hprt1*	0.259
*ef1a*	0.365	*gapdh*	0.708	*gapdh*	0.508	*gapdh*	0.287
*gapdh*	0.385	*ef1a*	−0.016	*ef1a*	0.668	*ef1a*	0.396

Abbreviations: SD = Standard deviation, r = Pearson product-moment correlation coefficient, SV = stability value, M = M value of stability.

### Candidate target gene expression

**Figure 2:**
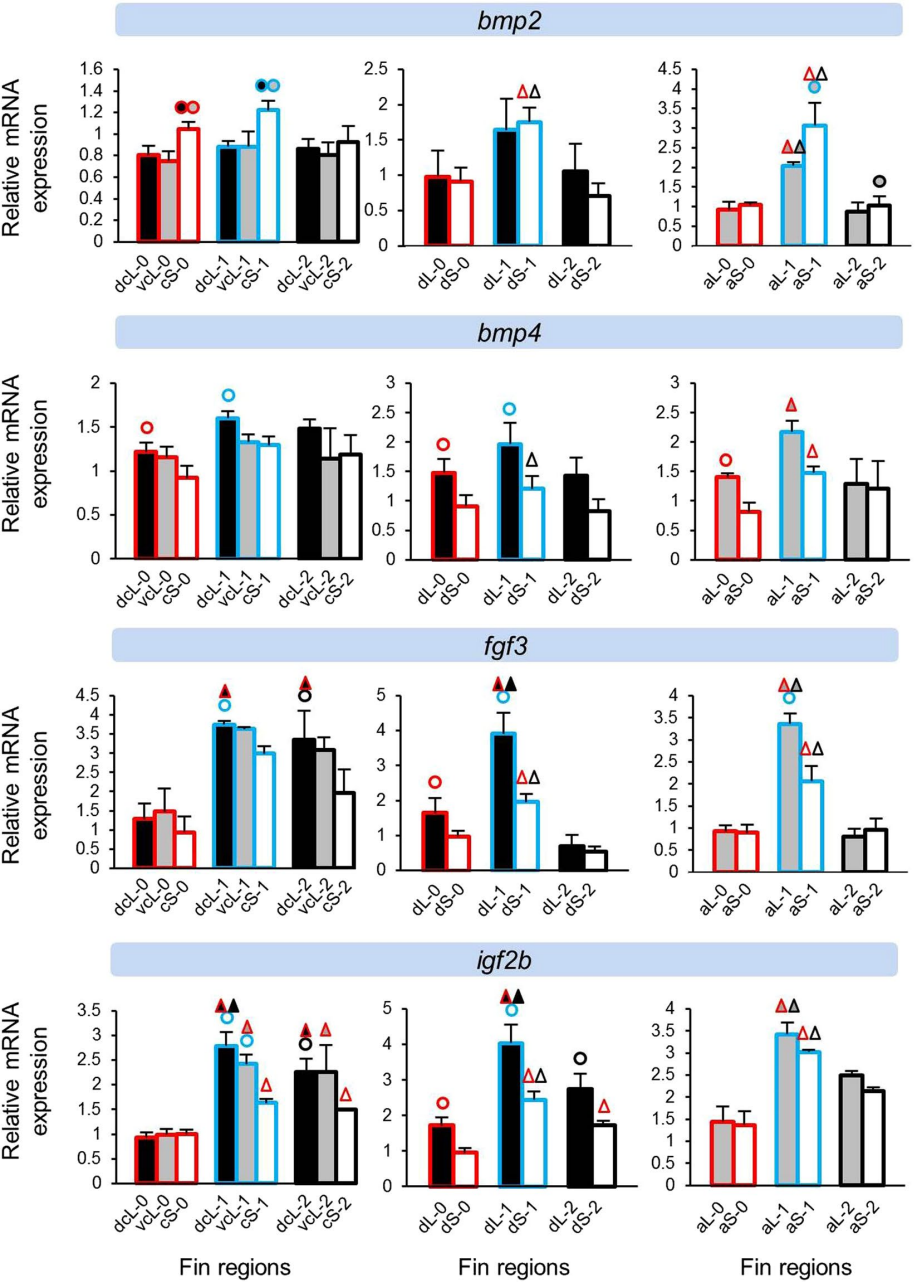
Expression levels of skeletogenic and growth factors *bmp2*, *bmp4*, *fgf3* and *igf2b*. Means and standard deviations of RQ in three biological replicates are shown for the elongated (L) and short (S) regions of the caudal, dorsal and anal fin in original (stage 0) and regenerating tissue. See Fig. 1A for fin region codes; numbers 0 to 2 identify regeneration stages. Circles above bars indicate significantly elevated expression (P < 0.05 in paired t-tests) in comparisons between L and S tissue samples (i.e., compared to the bar matching the colour code of the circle); note that the analysis was restricted to comparisons within the same fin type and the same regeneration stage. Triangles above bars indicate significantly elevated expression (P < 0.05 in paired t-tests) in comparisons among regeneration stages; comparisons were restricted to corresponding fin types and regions.

**Figure 3:**
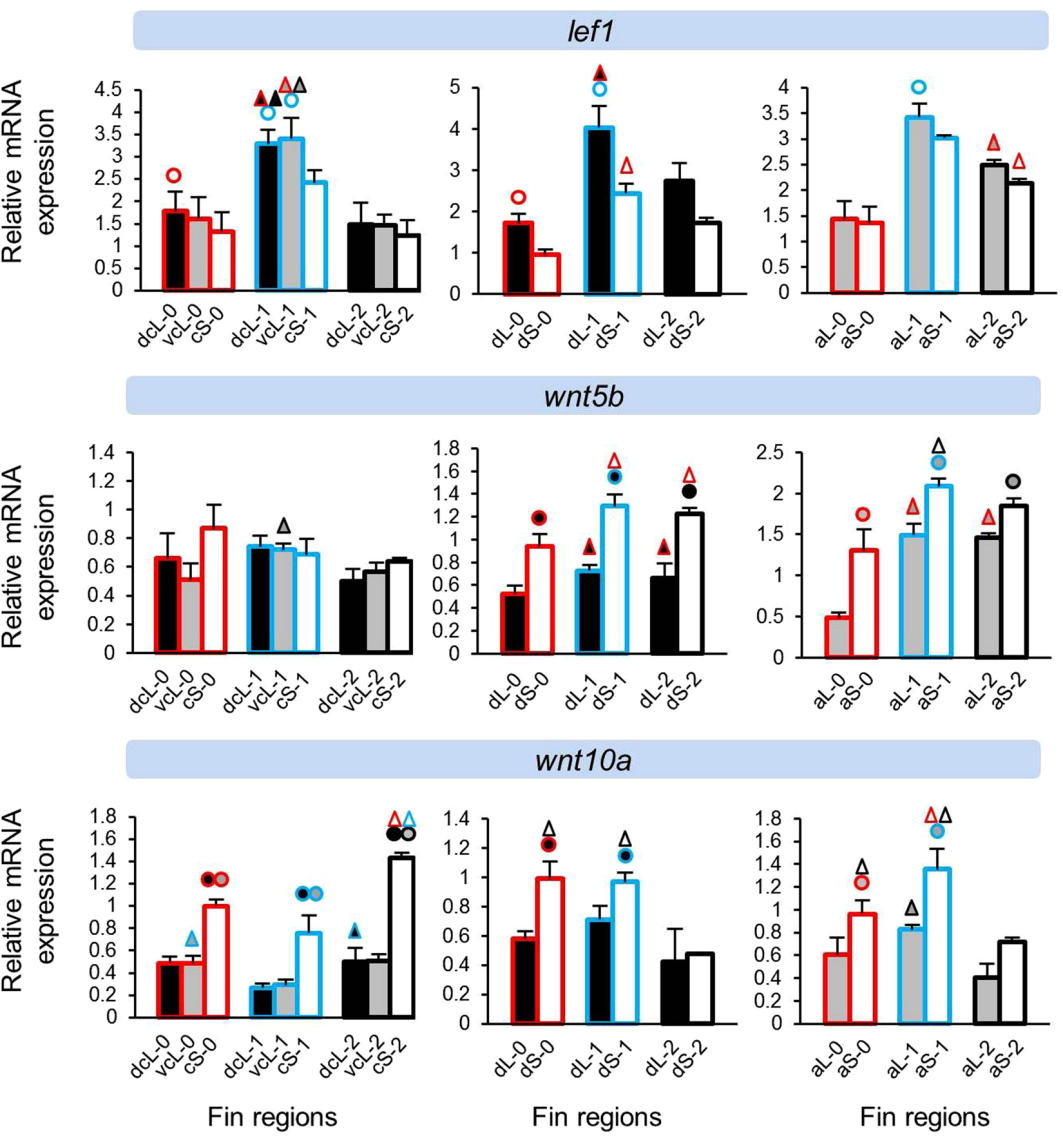
Expression levels of genes encoding components of WNT signalling (*lef1*, *wnt5b* and *wnt10a*). Means and standard deviations of RQ in three biological replicates are shown for the elongated (L) and short (S) regions of the caudal, dorsal and anal fin in original (stage 0) and regenerating tissue. See Fig. 1A for fin region codes; numbers 0 to 2 identify regeneration stages. Circles above bars indicate significantly elevated expression (P < 0.05 in paired t-tests) in comparisons between L and S tissue samples (i.e., compared to the bar matching the colour code of the circle); note that the analysis was restricted to comparisons within the same fin type and the same regeneration stage. Triangles above bars indicate significantly elevated expression (P < 0.05 in paired t-tests) in comparisons among regeneration stages; comparisons were restricted to corresponding fin types and regions.

**Figure 4:**
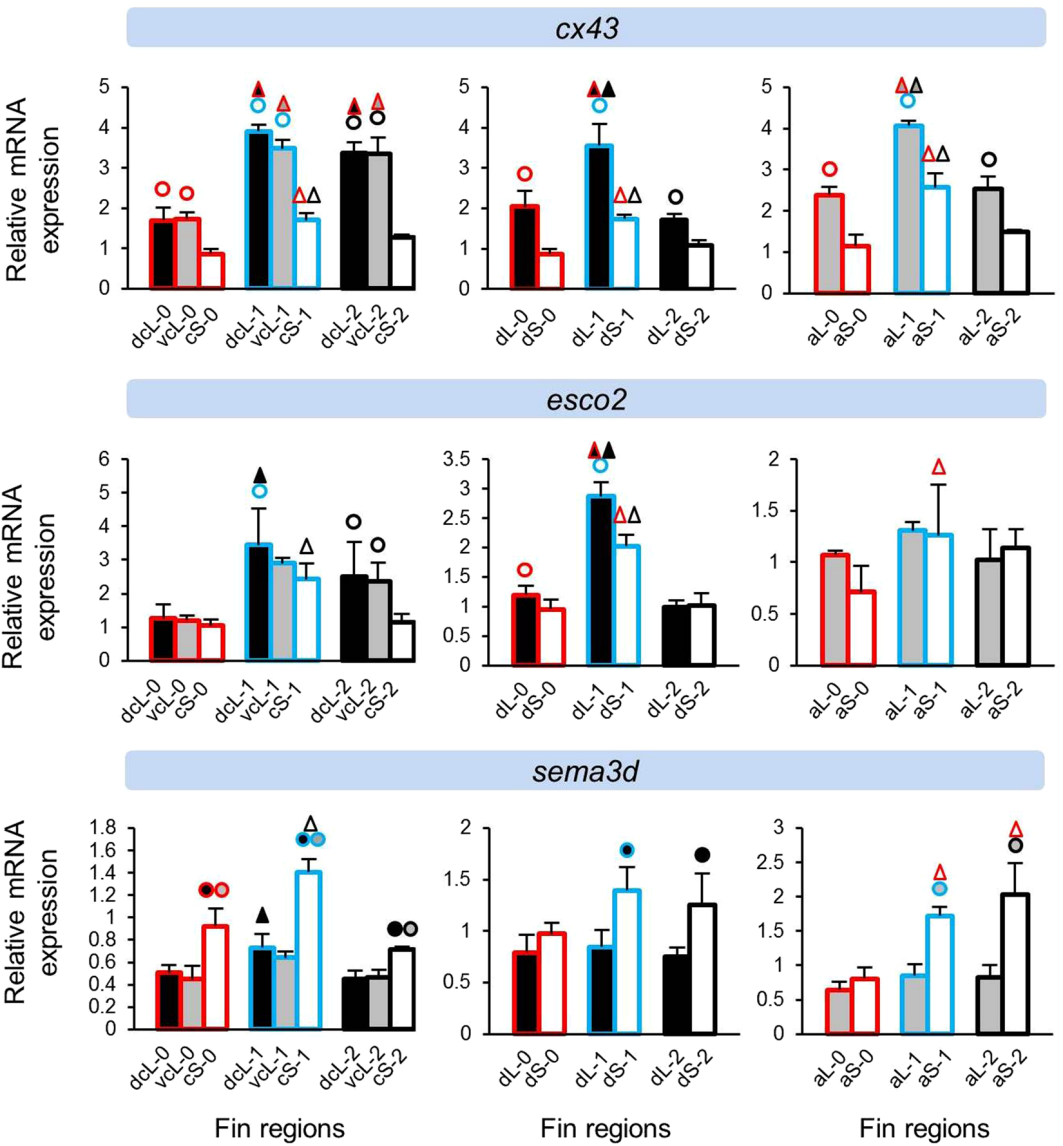
Expression levels of genes involved in fin ray formation and cell differentiation in the original and regenerating fin tissue (*cx43*, *esco2* and *sema3d*). Means and standard deviations of RQ in three biological replicates are shown for the elongated (L) and short (S) regions of the caudal, dorsal and anal fin in original (stage 0) and regenerating tissue. See Fig. 1A for fin region codes; numbers 0 to 2 identify regeneration stages. Circles above bars indicate significantly elevated expression (P < 0.05 in paired t-tests) in comparisons between L and S tissue samples (i.e., compared to the bar matching the colour code of the circle); note that the analysis was restricted to comparisons within the same fin type and the same regeneration stage. Triangles above bars indicate significantly elevated expression (P < 0.05 in paired t-tests) in comparisons among regeneration stages; comparisons were restricted to corresponding fin types and regions.

**Figure 5:**
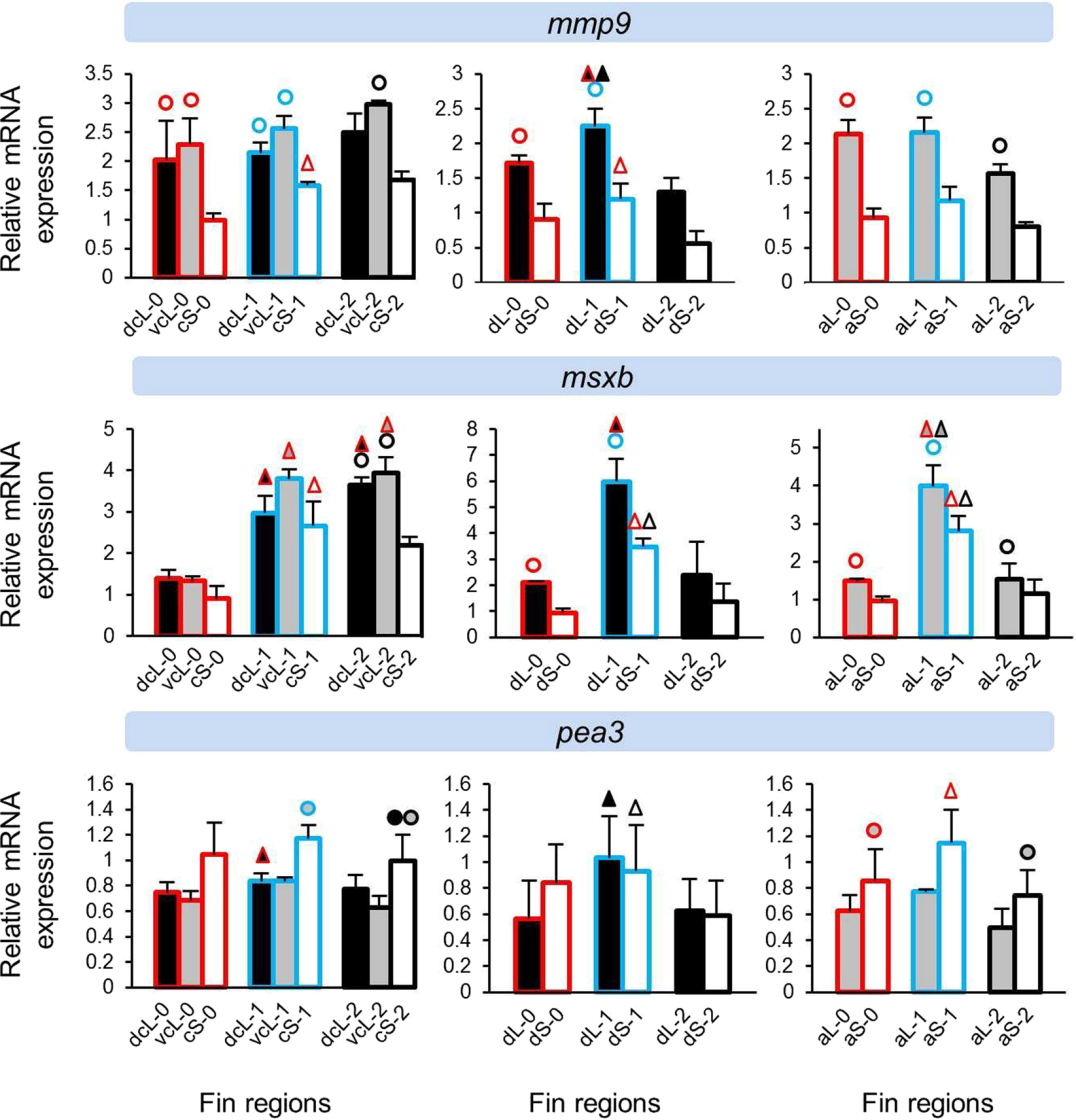
Expression levels of candidate genes *mmp9*, *msxb* and *pea3*. Means and standard deviations of RQ in three biological replicates are shown for the elongated (L) and short (S) regions of the caudal, dorsal and anal fin in original (stage 0) and regenerating tissue. See Fig. 1A for fin region codes; numbers 0 to 2 identify regeneration stages. Circles above bars indicate significantly elevated expression (P < 0.05 in paired t-tests) in comparisons between L and S tissue samples (i.e., compared to the bar matching the colour code of the circle); note that the analysis was restricted to comparisons within the same fin type and the same regeneration stage. Triangles above bars indicate significantly elevated expression (P < 0.05 in paired t-tests) in comparisons among regeneration stages; comparisons were restricted to corresponding fin types and regions.

Next, we assessed the expression levels of 40 candidate target genes in the elongated and short regions of the dorsal, anal and caudal fins. Gene expression was quantified in biopsies of the original fin tissue (stage 0) and at two stages during tissue regeneration. Since stage 0 tissue samples were taken from young adults (Supplementary Fig. 1), they represent growing fins. At regeneration stage 1 (day 15), the elongation of the fin tips had just become apparent, and at stage 2 (day 35), elongation was pronounced but fin regeneration not yet completed (Fig. 1B). Since fin types are known to regenerate at different speed^5^ and may also differ in their anatomical properties, comparisons of expression levels between elongated and short regions were carried out within each fin type separately (Supplementary data 2). We focus on 13 genes which showed significant expression differences between the elongated and short regions in at least two stages of two fins. For the presentation of the expression data, we classified these genes by their functional properties, *i*.*e*. growth and skeletogenic factors (Fig. 2), components of WNT signaling (Fig. 3), genes involved in fin ray segment formation (Fig. 4) and other genes with different functions (Fig. 5). Additionally, expression data for all of the investigated genes are shown in Supplementary data 2.

### Expression differences between short and long fin regions

Several patterns emerged in the comparisons of relative gene expression levels between elongated and short fin regions. ‘Expression in the elongated region’ is abbreviated as ‘L-expression’ in the following text, and reported as ‘higher’ or ‘lower’ in comparison to expression in the short region (‘S-expression’). We note that the design of our study only allowed for the detection of pronounced differences, such that non-significant contrasts do not necessarily imply the absence of an expression difference. The direction of significant expression differences detected between tissue samples (i.e. higher or lower L-expression) was consistent within genes. More genes were found with signals of higher L-expression (n = 8 genes; *bmp4*, *fgf3*, *igf2b*, *lef1*, *cx43*, *esco2*, *mmp9* and *msxb*) than lower L-expression (n = 5 genes; *bmp2*, *wnt5b*, *wnt10a*, *sema3d* and *pea3*) (Figs 2–5). Two genes (*cx43* and *mmp9*) showed higher L-expression in all fins and at most stages. Higher L-expression of *bmp4*, *fgf3*, *lef1* and *msxb* was detected in all fins, but not at all stages. Fin-specific differences were observed for *igf2b* and *esco2*, with higher L-expression only in caudal and dorsal fins. Lower L-expression across all fins and most stages was observed for *sema3d* and *wnt10a*. Interestingly, while *wnt10a* showed the strongest L/S expression differences in the caudal fin, lower L-expression of *wnt5a* was restricted to the dorsal and anal fins (Fig. 3). *bmp2* and *pea3* showed fin-specific lower L-expression (caudal and anal fins). Additionally, we note contrasting L/S expression differences for two skeletogenic genes, *bmp2* (lower L-expression) and *bmp4* (higher L-expression) (Fig. 1).

The gene expression analyses in the caudal fin consisted of two comparisons, (1) between the dorsal elongated (dcL) and the centre short (cS) region and (2) between the ventral elongated (vcL) and the cS region. For some genes, only one of these comparisons yielded a significant result. While these instances could represent dorso-ventral differences in gene expression levels, some of these genes showed dcL/cS and vcL/cS differences in the same direction, even though only one of the comparisons was significant (e.g. *mmp9* at stage 2; Fig. 5). When we compared dcL and vcL expression levels to test for dorso-ventral differences, significant contrasts were observed for only few genes (Supplementary data 2). We also note that for some genes, L/S expression differences fell below statistical significance at individual regeneration stages (e.g. *mmp9* at stage 2 in the dorsal fin; Fig. 5). We consider it likely that in some cases, the absence of consistent statistical test results in corresponding tissue comparisons can be ascribed to power limitations imposed by our study design.

### Expression differences across regeneration stages

Gene expression levels in the short and elongated regions of each fin were compared between the original and the regenerated biopsies (i.e. stage 0 versus stage 1 and 2) in order to detect up- or downregulation of genes during regeneration, in particular differences in gene regulation across regeneration between short and elongated fin regions. We focus on the results for the genes reported above (Figs 2–5); complete data are given in the Supplementary data 2. Again, the design of our study only allowed for the detection of pronounced differences. Most of the variation in gene expression across stages concerned the upregulation of genes in regenerating tissue compared to the original tissue, and was not specific to either the short or the elongated fin regions (e.g. *igf2b*, *msxb*, *cx43*, *fgf3*). For two genes, we detected significant upregulation in the short region of one of the fins, whereas expression did not change across stages in the elongated region (dorsal fin, *wnt10a*; anal fin, *sema3d*).

### Expression correlation analyses

The analysis of pairwise gene expression correlations can be an initial step towards identification of gene regulatory networks (GRNs) and coexpression modules for functional/transcriptional investigations^18^. In non-model teleost fish, analyses of gene expression correlations have frequently been used to identify GRNs potentially involved in morphological variation in skeletal structures^19–22^. In our study, pairwise comparisons of gene expression levels among the 13 genes with L/S expression differences revealed a large number of significant correlations. The majority of the significant results concerned positive correlations (blue shadings in Supplementary Fig. S2), many of which occurred in all three fins (framed cells) indicating potential co-regulatory mechanisms. Negative expression correlations (red shadings in Supplementary Fig. S2) were mainly restricted to the caudal fin. The strongest positive correlations detected in all fins for the genes with lower L-expression were observed between *wnt5b-sema3d* and *bmp2-pea3*. Strong correlations between genes with higher L-expression were found for, e.g., *esco2-msxb*, *msxb-lef1*, *igf2b-msxb-cx43*, *fgf3-msxb-bmp4-cx43*, and *cx43-mmp9*. Furthermore, *bmp4*, *cx43* and *msxb* showed the highest number of positive expression correlations with other genes across the three fins, while expression of *wnt5b* and *wnt10a* was correlated with few other genes. None of the negative correlations were observed across fins.

## Discussion

Given that ontogenetic and regenerative growth of fish fins depends on the expression of a vast number of genes^9^, it is plausible that some of these genes, belonging to different signaling pathways, are also involved in the determination of fin shape. Expression profiling studies in fish were mainly focused on the identification of genes that play a role in the general mechanisms of growth and regeneration of the caudal fin^6,10,23,24^. Hence, little is known about whether these gene regulatory networks determine fin shape traits, such as regional outgrowth, as well. In swordtail fish, where males grow a sword-like elongation on the ventral part of their caudal fin, sword-specific gene expression patterns were detected for a number of genes^14,25,26^.

We hypothesized that positional differences in gene expression levels underlie the extreme elongation of filaments at a specific region of a fin, as they occur in the unpaired fins of our study fish *N*. *brichardi*. Among the 40 candidate genes involved in zebrafish fin growth/regeneration that were screened for expression differences between elongated (L) and short (S) fin regions of *N*. *brichardi*, 13 genes showed repeated L/S expression differences. Most of these expression patterns were consistent with information on gene functions and interactions in the zebrafish, but we also noticed a few incongruences. Before discussing the 13 genes in more detail, we note that in our experiment, the elongated (L) and the short (S) tissue samples of the caudal fins also represent different positions along the proximo-distal axis (with L more distal than S), and L/S expression differences could therefore reflect expression gradients along this axis. A recent study described proximo-distal gene expression gradients along the zebrafish caudal fin for a number of genes^11^. None of the genes discussed on our study appeared in the ‘high confidence’ list of differentially expressed genes that was assembled from RNAseq and proteomics data, but some (*bmp2*, *bmp4*, *lef1*, *wnt5b*, *sema3d*, *mmp9*, *igf2b*) overlapped with the results based on RNAseq alone (Dataset S3 in Rabinowitz *et al*. 2017)^11^. However, the direction of the L/S difference in *N*. *brichardi* matched the proximo-distal gradient in zebrafish in only two cases (*bmp4* and *lef1*). Unless proximo-distal expression gradients differ widely between zebrafish and *N*. *brichardi*, we may assume that our L/S comparison was not confounded by position along the proximo-distal axis.

Furthermore, the margins of the fins of *N*. *brichardi* are coloured white (Supplementary Fig. S1A), indicating the presence of light-reflecting iridophores and/or white leucophores, and expression differences between elongated and short regions could reflect different chromatophore compositions. We reduced this problem by using young adults, in which the white coloration is still less pronounced than in fully grown individuals, and removed most of the white margins from the tissue sample (Supplementary Fig. S1B). None of the genes showing expression differences between the long and short fin regions are enriched in the iridophore transcriptome of zebrafish^27^. However, since little is known about leucophore specific genes^28^, we cannot assess whether the possible presence of leucophores in elongated fin regions might contribute to the identified gene expression differences in this study.

Among the tested skeletogenic and growth factors, we found increased L-expression of genes encoding a fibroblast growth factor, *fgf3*, and an insulin growth factor, *igf2b*, in regenerating fins (Fig. 2). Both factors are known to be required for caudal fin regeneration in zebrafish through induction of the blastemal and wound epidermal markers (by igf2b) and promoting cell proliferation in the blastema (by fgf3)^29,30^. Intriguingly, the insulin/insulin-like pathway (ILS) has been proposed as a mechanism for tissue-specific exaggerated growth, linking the development of ornaments and weapons to individual condition^31^. Furthermore, a possible role of FGF signalling in fin shape determination has been demonstrated in the swordtail fish, where the growth of the sword-like extension of the male caudal fin involved the activation of the FGF pathway^25^. Interestingly, two genes coding for bone morphogenetic proteins (BMPs), *bmp2* and *bmp4*, displayed opposite L/S expression differences, though the pattern was not repeated across all fins and stages (Fig. 2). BMP mediated signals are involved in induction of osteoblast differentiation and skeletogenesis during fin development and regeneration^23,32–35^. In the zebrafish caudal fin, expression of *bmp4* is restricted to the distal blastemal, whereas *bmp2* is expressed in newly differentiating scleroblasts and in the adjacent cells of the basal layer of the epidermis^34^. Possibly, the higher L-expression of *bmp4* in our study may reflect elevated cell proliferation activity in the elongated fin regions, whereas higher S-expression of *bmp2* may be due to a shift towards bone differentiation in most of the short fin tissue.

WNT/β-catenin signaling is known for its pivotal role in regulating different aspects of fin development and regeneration such as establishment of the distal blastema and specification of the lateral basal epidermal layer^8,9,32,36–39^. WNT signaling functions through complex interactions with other signals including BMP, Hedgehog (HH) and FGF pathways, and different WNT components can have opposing effects during epimorphic fin regeneration^32,37,38^. Notably, we found lower L-expression of two WNT ligands, *wnt5b* and *wnt10a*, across original and regenerating tissue of *N*. *brichardi* fins (Fig. 3). The decreased L-expression of *wnt5b* was in agreement with the findings in zebrafish where its overexpression reduced proliferation of the blastema mesenchyme and the overlying epithelium in the regenerating fin^37,38^. However, the decreased L-expression of *wnt10a* was in contrast to the zebrafish findings, since a *wnt10a* activated β-catenin-dependent signal is required for blastema formation and subsequent regeneration^37^. It should be noted that wnt5b activates a non-canonical WNT signalling pathway, but can also negatively regulate the canonical WNT signaling pathway during fin regeneration^37^. Importantly, the expression patterns of *wnt5b* and *wnt10a* displayed different profiles between the fins, *i*.*e*. while *wnt5b* L/S expression differences were detected only in the anal and dorsal fins, *wnt10a* had the most pronounced L/S expression differences in the caudal fin. This could indicate fin and/or axis specific functions of these ligands.

Another interesting set of target candidates encompassed components of a gene regulatory network (*i*.*e*. *cx43*, *esco2*, *sema3d*) involved in formation, growth and regeneration of fin ray segments and joints^40–44^. Two of these genes, *cx43* and *sema3d*, displayed L/S expression differences indicating distinct activation of the network in these fin regions (Fig. 4). *cx43*, a gene encoding a subunit of the gap junction protein complex by which neighbouring cells exchange small molecules, was of particular interest because of its known role in regulation of fin ray length in zebrafish^40^ and its consistent higher L-expression in the present study. The length of fin rays depends on the number and size of bony segments^8^ and reduced expression of *cx43* in zebrafish *sof* mutants has been shown to cause defects in the lengthening of bony fin ray segments^40^. The same study has identified *cx43* expression in both the germinal region of newly growing segments and in the osteoblasts at segment boundaries and joints^40^. Further investigation in zebrafish revealed that overexpression of *cx43* suppresses segment joint formation and increases cell proliferation resulting in elongated bony fin rays^45,46^. Opposite to the findings in zebrafish, increased expression of *cx43* in L fin regions of *N*. *brichardi* was not accompanied by increased length of fin ray segments (Fig. 1C). On the contrary, we found a slight increase in segment length in S regions compared to L regions. This indicates that, first, the elongation of L regions does not involve increased length of fin ray segments. Secondly, overexpression of *cx43* in L regions might also involve other mechanism(s), distinct from the one causing fin ray elongation in zebrafish, during fin morphogenesis in *N*. *brichardi*.

A possible reason for the discrepancy between zebrafish studies and our results could be the increased expression of *sema3d* in short fin regions, a gene encoding a conserved secreted ligand activating several cell surface receptors, which acts downstream of cx43 and mediates cx43-dependent cell proliferation and joint formation in zebrafish^47^. We found positive expression correlations between *sema3d* and *wnt5b* across all three fin types. This pattern could potentially be due to a regulatory link between these genes, and certainly invites further investigations. In contrast, the absence of an expression correlation between *cx43* and *sema3d* and even contrasting L/S expression across the fins could suggest their regulatory decoupling and potential involvement of a distinct upstream effector for *sema3d* during fin morphogenesis in *N*. *brichardi*. It is worth emphasizing that although the expression levels of *cx43* and *sema3d* appeared to be correlated in the zebrafish caudal fin, *sema3d* was not a direct target of cx43 and had a distinct expression pattern than *cx43* during fin regeneration^47^. A recent study in zebrafish has demonstrated esco2, an enzyme establishing chromatid cohesion in mitosis and required for craniofacial and limb development in human, to be an upstream transcriptional regulator of *cx43* and *sema3d* ^44^. In our results, except for *cx43* in caudal and dorsal fins, we did not find consistent expression correlation between *esco2* and these genes. We also note that, while *cx43* downregulation was associated with upregulation of *mmp9* in the zebrafish caudal fin^46^, both *cx43* and *mmp9* were more strongly expressed in L than in S tissue in our study. Such discordances between gene function in zebrafish and expression patterns observed in *N*. *brichardi* may be suggestive of divergence in regulatory mechanisms between taxa.

Finally, the other candidates showing differential L/S expression include a cell differentiation factor, *msxb*, a member of matrix metalloproteinase family, *mmp9*, and *pea3*, an Ets-related transcription factor and downstream target of FGF signalling (Fig. 5). In zebrafish, *msxb* regulates cell proliferation during caudal fin regeneration and is activated by FGF signalling^48^, which is consistent with the expression patterns observed in our study. Pronounced expression differences were observed for *mmp9* with higher L-expression across all the fins and most stages and in fact *mmp9* also showed a strong positive expression correlation with *cx43* in all fins. In addition, the higher L-expression of both *mmp9* and *cx43* in the intact fins (day 0) could indicate their role in maintenance of the phenotype in adult *N*. *brichardi*. In the zebrafish caudal fin, a reduction of *cx43* expression led to increased expression of *evx1* and *mmp9* (which acts downstream of *evx1*)^46^. This regulatory connection is not reflected in our observations, as *evx1* showed no L/S expression differences, whereas both *cx43* and *mmp9* were more strongly expressed in the L region. Again, components of the regulatory pathway may have diverged between the taxa. Even within the zebrafish, different alleles of *cx43* have been shown to cause different phenotypes^49^.

The existence of upstream regulator(s)/signaling pathway(s) that directly regulate both *cx43* and *mmp9* independent of *evx1* is another potential scenario, suggesting that the identification of shared upstream regulator(s) of *mmp9* and *cx43* during fin growth and regeneration would be a promising agenda for future studies. The extracellular proteinase encoded by *mmp9* participates in ECM remodelling by degrading specific types of collagens and has been shown to be highly expressed in populations of epidermal and mesodermal cells in regenerating fins^10^. In zebrafish, inhibition of mmp9 function during larval fin fold regeneration severely impaired this process through perturbing the reconfiguration of tissues (e.g. collagen fiber reorganization) but not cell proliferation and migration^10,50^. Furthermore, *mmp9* is a member of a conserved co-expression gene network which acts downstream of several signaling pathways involved in craniofacial skeletal morphogenesis^21^. In contrast, little is known about its upstream regulator(s)/signaling pathways during fin morphogenesis. To our knowledge, the only signaling pathway shown to act upstream of *mmp9* during fin regeneration is a signal transduced by aryl hydrocarbon receptor (AhR) which mediates the effects of a wide array of exogenous chemicals in different tissues^51^. The same pathway has been shown to disrupt cell communication through suppression of *cx43* expression in human tissues^52^, though this has yet to be confirmed in regenerating fish fin. Interestingly, a receptor of the AhR pathway (*ahr1*) has a conserved genomic location close to *evx1* gene in zebrafish and human, which might indicate a conserved functional and/or transcriptional link between the two genes^53^. Furthermore, activation AhR pathway has been shown to induce *evx1* expression during zebrafish development^54^.

## Conclusions

Our analysis identified expression differences between short and elongated fin regions in approximately one third of the tested candidate target genes. The differently expressed genes belong to different molecular pathways, including WNT, BMP, ILS and FGF signalling, as well as a regulatory network involved in fin ray growth and regeneration. Expression patterns were not fully concordant across fins, which is an important finding considering the almost exclusive focus on the caudal fin in previous studies of fin regeneration. Moreover, some of the observed patterns were not consistent with expression correlations and mutant phenotypes described in the zebrafish model. Fin- and taxon-specific patterns suggest that regulatory mechanisms are not uniform across unpaired fins and have diversified across taxa. Finally, the validation of stably expressed reference genes for the specific study conditions (species, tissue and laboratory protocol) is a technical asset of our study, which we recommend to incorporate in all qPCR analyses. While the expression differences observed in our study point us to genes that might be involved in the exaggerated growth of the elongated fin filaments, we cannot rule out the possibility that some signals also reflect axial patterns or concordant histological (e.g. pigmentation) differences between the fin regions. The phenotypic diversity among cichlids offers opportunities to address potentially confounding effects. For instance, genes identified in the present study can be tested in Lamprologini species with differently shaped fins and with differently coloured fin elongations for consistency with their role in regional fin outgrowth. The present study represents a first step towards the elucidation of the molecular mechanisms behind fin shape variation in cichlid fishes and lays the foundation for continuative studies.

## Methods

### Fin sampling and measurement of segment length

Fin biopsies were obtained from 24 captive bred young adult individuals of *N*. *brichardi* (12 males and 12 females) sized 5-7 cm (total length) and with intact fins. The elongated regions in unpaired fins of *N*. *brichardi* develop in juveniles of both sexes without any apparent sexual dimorphism in shape and length. Prior to the experiment, the fish were housed in pairs in twelve separate aquaria and fed on identical diets for one month. To obtain fin tissue biopsies, the fish were anesthetized in water containing 0.04 gram per litre of MS-222, and their dorsal, anal, and caudal fins were cut in front of the first ray bifurcation (branching) under a stereomicroscope (red dashed lines in Fig. 1A,B). Separate tissue samples were then taken from the elongated and the short region of each fin biopsy (green and yellow areas in Fig. 1A). Tissue samples representing elongated fin regions contained the last 6-7 fin ray segments of two elongated fin rays (red arrows in Supplementary Fig. S1B) and the connecting inter-ray tissues. Likewise, tissue samples representing the short fin regions contained 6-7 segments of two short fin rays (blue arrows in Supplementary Fig. S1B) and the connecting inter-ray tissues. In order to reduce a confounding effect of the accumulation of iridophores and/or leucophores along the margins of the elongated fin regions (white margins visible in Supplementary Fig. S1A), we did not include the fin rays at the edges of fins (green arrows in Supplementary Fig. S1B) in our samples. Each tissue sample was immersed in RNAlater (Qiagen) and stored frozen until RNA isolation.

We quantified gene expression in the original tissue (stage 0) and during regeneration. Therefore, we performed a second biopsy 15 days after the first cut, when the elongation of the fin tips had just become apparent (stage 1), followed by a third biopsy after 35 days, when elongation was pronounced but fin regeneration not yet completed (stage 2; Fig. 1B).

Throughout the paper, the investigated tissues types are identified by fin type (dorsal, caudal, anal), region (elongated, short) and stage (0, 1, 2), such that, for instance, aS-0 stands for the original tissue of the short region of the anal fin. In the caudal fin, we distinguish between the dorsal and the ventral elongated regions, such that dcL stands for the dorsal elongated region of the caudal fin, vcL for the ventral elongated region, and cS identifies the short region (Fig. 1A).

To compare the length of fin ray segments between elongated and short regions, we stained fin samples from 4 fish (two males and two females) with alizarin red using a modification (8 days clearing in 10% KOH) of the acid-free double staining protocol described by Walker and Kimmel (2007)^55^. Photographs taken with an Olympus OM-D EM-5 II camera, attached to Olympus SZX-ILLD2-200 binocular, were imported into ImageJ, where we measured the length of the five most distal, complete segments of two branches of the shortest and the longest rays per fin (or, for the caudal fin, of two branches of the longest dorsal and ventral rays). To account for possible correlations within rays and individual fins, linear mixed models (R package nlme, with ‘segment’ nested in ‘ray’ as grouping factors)^56^ were used to compare segment length between the elongated and short regions of each fin type.

Anaesthesia and fin biopsies were performed under permit number BMWFW-66.007/0024-wF/v/3b/2016 issued by the Federal Ministry of Science, Research and Economy of Austria (BMWFW). All methods were performed in accordance with the relevant guidelines and regulations of BMWFW.

### RNA isolation and cDNA synthesis

Corresponding tissue samples from 8 fish (four males and four females) were pooled as biological replicates (n = 3 replicates) and transferred to tubes containing TRI Reagent (Sigma) and 1.4 mm ceramic spheres. The samples were homogenized by FastPrep-24 Instrument (MP Biomedicals, Santa Ana, CA, USA). RNA was extracted according to manufacturer’s Trizol protocol and dissolved in 40 µl RNase-free water. RNA samples were treated with DNase (New England Biolabs) to remove contaminating DNA. RNA concentration was measured by spectrophotometry using a Nanophotometer (IMPLEN GmbH, Munich, Germany). The quality of the RNA samples was evaluated in a R6K ScreenTape System on an Agilent 2200 TapeStation (Agilent Technologies) to ensure that the integrity number (RIN) of all samples was higher than 7. cDNA was prepared from 1000 ng of RNA using the High Capacity cDNA Reverse Transcription kit (Applied Biosystems), according to the manufacturer’s protocol. Negative controls, i.e. reactions without addition of reverse transcriptase (-RT samples), were prepared to confirm the absence of genomic DNA. cDNA was diluted 1:3 times in nuclease-free water for further use in quantitative real-time PCR.

### Gene selection, Primer design and real-time qPCR

To validate suitable reference genes for expression analysis, we screened 7 genes expressed in a variety tissues and frequently used as reference genes for qPCR studies of body compartments and skeletal structures in teleost fishes (Supplementary data 1)^17,57–59^. Additionally, we selected 40 candidate target genes that are known to be involved in fin development, morphogenesis and/or regeneration based on findings in zebrafish (Table 1 and Supplementary data 1).

The qPCR primers were designed based on recently released transcriptome data of *Neolamprologus brichardi* ^15^. The sequence alignment was performed using CLC Genomic Workbench, version 7.5 (CLC Bio, Aarhus, Denmark) and locations overlapping the exon boundaries of the genes were determined by the Nile Tilapia annotated genome sequences in the Ensembl database (http://www.ensembl.org/Oreochromis_niloticus). The qPCR Primers were designed on exon boundaries using Primer Express 3.0 software (Applied Biosystems, Foster City, CA, USA) and checked for self-annealing, hetero-dimers and hairpin structures with OligoAnalyzer 3.1 (Integrated DNA Technology) (Supplementary data 1).

Real-time PCR was performed in 96 well-PCR plates on an ABI 7500 real-time PCR System (Applied Biosystems) using Maxima SYBR Green/ROX qPCR Master Mix (2X) as recommended by the manufacturer (Thermo Fisher Scientific, St Leon-Rot, Germany). Each biological replicate was run in duplicate for each gene and the experimental set-up per run followed the preferred sample maximization method^60^. The qPCR was run with a 2 min hold at 50 °C and a 10 min hot start at 95 °C followed by the amplification step for 40 cycles of 15 sec denaturation at 95 °C and 1 min annealing/extension at 60 °C. A dissociation step (60 °C – 95 °C) was performed at the end of the amplification phase to identify a single, specific product for each primer set (Supplementary data 1). Primer efficiency values (E) were calculated with the LinRegPCR v11.0 programme (http://LinRegPCR.nl)^61^ and primer-pairs with E less than 0.9 were discarded and new primers designed (Supplementary data 1).

### Data analysis

To identify the most stably expressed reference genes, three ranking algorithms were used; BestKeeper^62^, NormFinder^63^ and geNorm^64^. The standard deviation (SD) based on Cq values of the fin regions was calculated by BestKeeper to determine the expression variation for each reference gene. BestKeeper also determines the stability of reference genes through a correlation calculation or BestKeeper index (r). GeNorm calculates mean pairwise variation between each gene and other candidates (the expression stability or *M* value) in a stepwise manner and NormFinder identifies the most stable genes (lowest expression stability values) based on analysis of inter- and intra-group variation in expression levels^17,58^.

The mean Cq values of the two top-ranked reference genes was used as Cq _reference_ and the difference between Cq values (ΔCq) of the target genes and the selected reference gene was calculated for each target gene; ΔCq _target_ = Cq _target_ − Cq _reference_. To obtain a ΔΔCq value, samples were normalized to the ΔCq value of a calibrator sample (ΔCq _target_ − ΔCq _calibrator_). To this aim, one arbitrarily chosen biological replicate of dL-0, aL-0 and dcL-0 was used to calibrate samples of the dorsal, anal and caudal fin, respectively. Relative expression quantities (RQ) were calculated based on the expression level of the calibrator sample (E^−ΔΔCq^)^65^. For each target gene, differences in gene expression levels between elongated and short fin regions as well as among regeneration stages were tested by paired t-tests on log-transformed RQ data. To assess similarities in the expression patterns of the target genes, Pearson correlation coefficients (*r*) were calculated in R (http://www.r-project.org).

### Data availability

All the data represented in this study are provided within the main manuscript or in the Supplementary materials.

### Ethical approval

All experimental protocols related to the fishes used in this study were approved by the Federal Ministry of Science, Research and Economy of Austria. Please identify the approving body and license numbers in the methods section.

## Electronic supplementary material

Supplementary Figures

Supplementary data 1

Supplementary data 2
