# Supplementary material for: A gene expression study of ornamental fin shape in Neolamprologus brichardi, an African cichlid species
Source: Sci Rep. 2017 Dec 12;7:17398. doi: 10.1038/s41598-017-17778-0 (PMC5727040; doi:10.1038/s41598-017-17778-0)
Supplement: Supplementary file 1 — Supplementary Figures [file 41598_2017_17778_MOESM1_ESM.pdf]

A gene expression study of ornamental fin shape in *Neolamprologus brichardi*, an African cichlid species

Ehsan Pashay Ahi[1],
Email: ehsan.pashay-ahi@uni-graz.at

Florian Richter[1]
Email : florianrichter1@outlook.com

Kristina M. Sefc[1],
Email: kristina.sefc@uni-graz.at

1. Institute of Zoology, University of Graz, Universitätsplatz 2, A-8010 Graz, Austria

Corresponding Author: Ehsan Pashay Ahi,
Email: ehsan.pashay-ahi@uni-graz.at

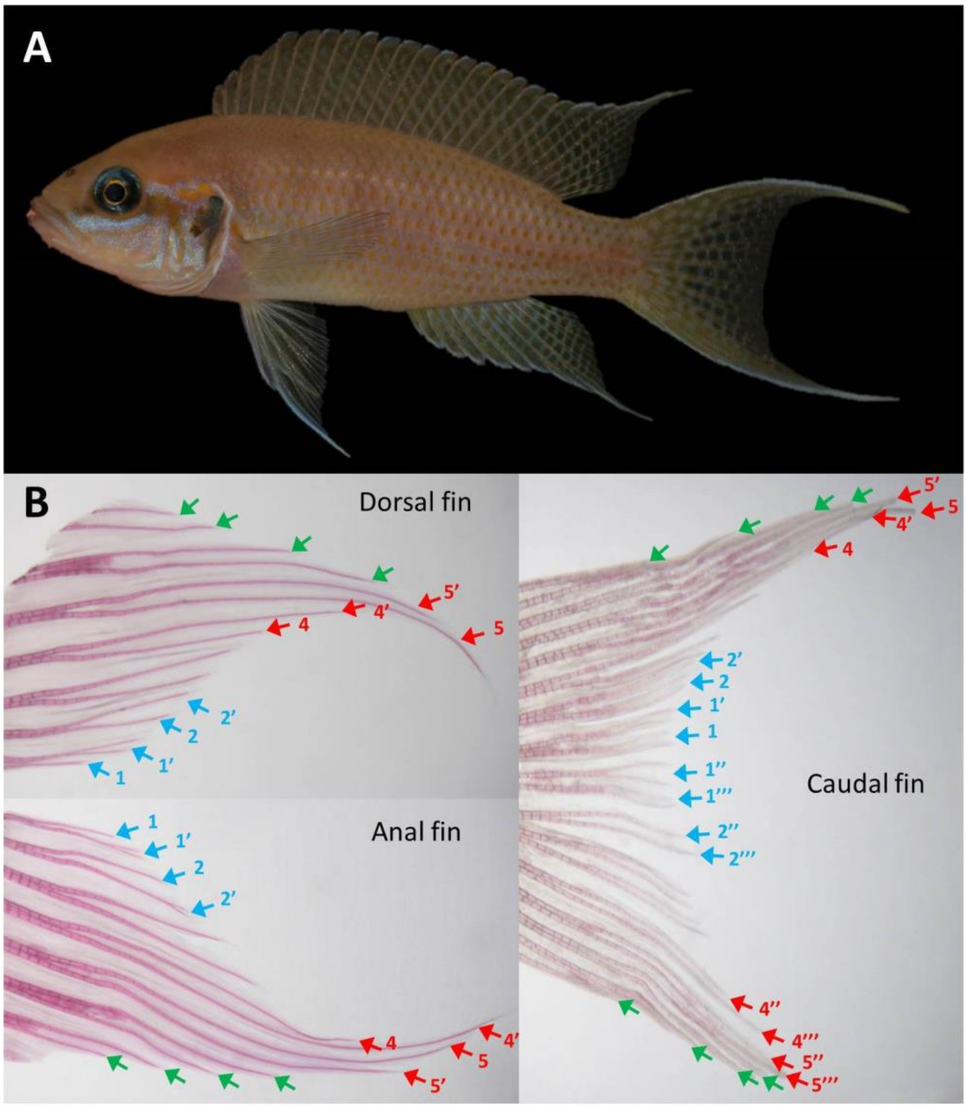

Supplementary Figure 1. The unpaired fins of *Neolamprologous brichardi*. (**A**) An adult *N. brichardi*, prior to fin amputation (**B**) Alizarin red stained fin rays, red and blue arrows specify rays included in the tissue samples of the elongated and short fin regions, respectively, of the anal, dorsal and caudal fin. Green arrows specify the fin rays situated in the white-coloured, leucophore- or/and iridophore-rich margins of the fins, which were removed from the tissue samples prior to RNA extraction in order to avoid confusion with colour-specific expression patterns.

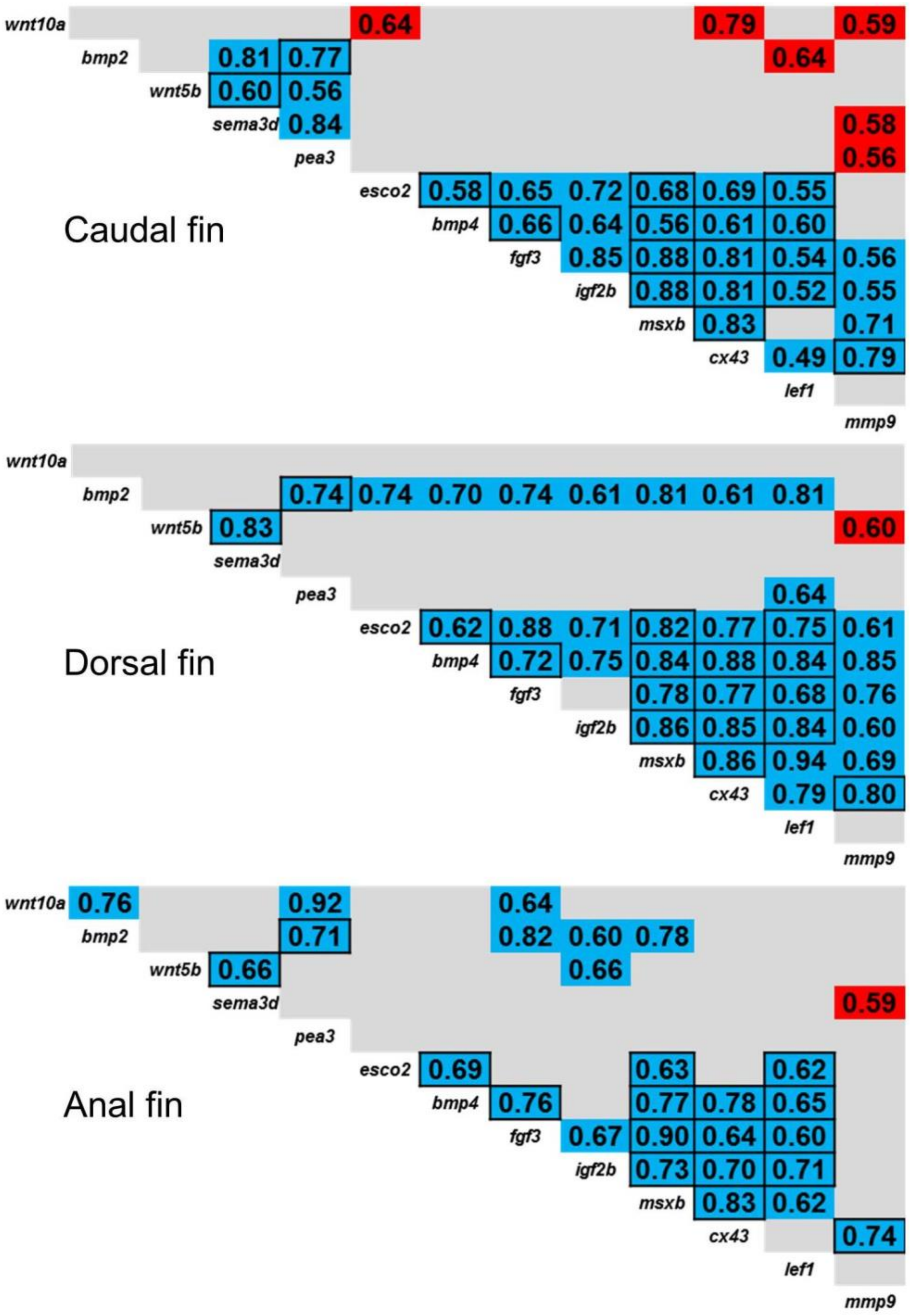

Supplementary figure 2. Significant expression correlations between the 13 focus target genes. Numbers indicate Pearson correlation coefficients ($P < 0.01$ in 2-tailed tests). Blue shading represents positive and red shading represents negative expression correlation.
